# Prevalence of Vitamin B12 Deficiency in Patients with Type II Diabetes Mellitus on Metformin: A Study from Khyber Pakhtunkhwa

**DOI:** 10.7759/cureus.1577

**Published:** 2017-08-18

**Authors:** Adnan Khan, Ihtesham Shafiq, Mohammad Hassan Shah

**Affiliations:** 1 House Officer, Rehman Medical Institute, Peshawar; 2 Rehman Medical Institute, Peshawar

**Keywords:** diabetes mellitus, metformin, b12 deficiency

## Abstract

Background

Metformin is the most common oral hypoglycemic used and associated with certain abnormalities. The objective was to evaluate and define the occurrence and bases of vitamin B12 deficiency amongst patients on Metformin for diabetes mellitus type II.

Methods

A cross-sectional study was conducted on 209 patients having diabetes type II between January-December 2016. The patients aged > 45 years and who had taken metformin for at least three months were recruited with regular follow-up at the Endocrinology Unit of Hayatabad Medical Complex and Diabetic Center Hayatabad, Peshawar. The patients were included in a survey after which they had their serum B12 levels measured. Serum B12 levels < 150 pg/ml is defined as the B12 deficiency.

Results

About 29.66% of diabetic patients had confirmed the B12 insufficiency through laboratory tests. The patients on metformin had statistically lower values of B12 (P = 0.01). For the patients who smoked, vitamin B12 deficiency was significantly higher than those who did not smoke (p= <0.001). Also in patients using multivitamins, vitamin B12 deficiency was lower compared to nonusers (p=0.05).

Conclusion

Our study shows that for the patients with type 2 diabetes (T2DM), long-term treatment with metformin and smoking are associated with higher chances of developing vitamin B12 deficiency. Clinicians should, therefore, recognize this significant element and should screen diabetics who are on metformin treatment for any B12 insufficiency, which may be hidden, especially patients coming with neurologic symptoms. Additionally, multi vitamins taken daily may have a protective role.

## Introduction

Diabetes mellitus affects more than 6% of the United States population, with the majority of the patients having type 2 diabetes mellitus (DM) [1]. During the past decade, an increase of 30% in the prevalence of DM has been recorded in the United States, dramatically in younger individuals. The frequency of diabetes mellitus in Pakistan is estimated to be about 7.7% in rural areas and about 10.6 % in urban areas while 7.2 million and higher individuals are affected by this ailment [2].

Metformin has been one of the most extensively used anti-diabetic agents taken orally. Metformin is the foundation of medicine in the treatment of non-insulin-dependent diabetes mellitus/ type II diabetes mellitus (NIDDM, T2DM) with approximations that it is frequently approved and recommended to 120 million patients with diabetes globally [3]. The majority of the side effects due to metformin is mild and usually include gastrointestinal symptoms, such as abdominal distress, soft stools, and diarrhea [4]. Generally, these adverse effects start shortly after the commencement of metformin and in time disappear after cessation of the drug.

Amassing evidence from observational along with interventional studies has shown the relation amongst prolonged usage of metformin and vitamin B12 deficiency. It may affect the calcium-dependent absorption of B12 [5]. The serum vitamin B12 values have been stated to be inversely related to the dose and duration of metformin usage [6-7].

Irrespective of the established association between metformin and vitamin B12 deficiency, the true problem has not yet been accurately quantified. Prior studies have indicated that the occurrence of vitamin B12 deficiency due to metformin differed immensely and ranged between 5.8% and 52% [5, 7-8].

The extended use of metformin, accompanied by vitamin B12 deficiency, may lead to increasing the considerable problem of peripheral neuropathy in non-insulin-dependent diabetes mellitus (NIDDM) patients. Neuropathy, being an impending health abnormality occurring due to vitamin B12 deficiency affects around 30% diabetics who are over 40 years of age and state about having a diminished sensory perception in their feet [9]. Regrettably, symptoms and signs of both diabetic neuropathy and paresthesia are somewhat similar, reduced vibration sense and diminished proprioception (vibration sense) linked to vitamin B12 deficiency [10]. Several studies conducted lately vexed to explain the possible relationship among prolonged metformin usage and its vitamin B12 deficiency associated peripheral neuropathy with contradictory results.

Furthermore, it seems challenging to confront the problem over randomized controlled trials as the necessary study duration, sample size and ethical issues make the use of such designs unfeasible. Currently, all the existing evidence has been derived from observational studies.

No specific literature exists in the Pakistani population, hence, a cross-sectional research study was conducted for outlining the occurrence of vitamin B12 deficiency among patients taking metformin for Type II Diabetes Mellitus (T2DM) to assess the causes linked with vitamin B12 deficiency occurring in the patients taking metformin.

## Materials and methods

Between January-December 2016, patients with type II diabetes, aged more than 45 years, were recruited at Endocrinology Unit, Medical Complex and Diabetic Center Hayatabad, Peshawar, Pakistan.

We acquired a well-versed approval for the study and requested all the subjects meeting the criteria, for inclusion in the study to complete the survey, which inquired patient biodata, medicinal use, and any added multivitamin supplement use.

The patients who were excluded from the study included those having a prior history of pernicious anemia, the chronic kidney disease which was well-defined by a creatinine value of > 3.0, previous surgery for weight loss, gastrectomy, previous gut resection or inflammatory bowel disease (especially Crohn’s disease). Medicines and additional multi-vitamin supplements inquired included metformin, insulin, added antidiabetic drugs, acid blockers (H2 blockers and/or proton pump inhibitors), herbal enhancements and B complex vitamins. Biodata inquired included age, gender, and duration since onset of diabetes or its diagnosis.

Data collection

Blood samples containing venous blood were acquired under completely sterile conditions. These samples were then stored under -30°C in secure tubes which were held upright. These samples were investigated on the same day for the measurement of vitamin B12 levels with a DXI automated analyzer. The measurement of glycosylated hemoglobin (HbA1c) was carried out via DXC-600 automated analyzer. The values obtained were then verified by a pathologist. The vitamin B12 levels less than 150 pg/mL was regarded as insufficient while vitamin B12 levels higher than 350 pg/mL was regarded as normal levels. Every patient’s profile included biodata, serum hemoglobin level, time since onset of diabetes, the dosage of metformin intake and time for the use of metformin. Every bit of data was stored in a Performa designed beforehand.

Statistical analyses

Entire data were stored in the Statistical Package for Social Sciences, SPSS (version 23.0) (IBM Corp., Armonk, New York, United States of America). Descriptive data were spread out in order to recapitulate. Mean along with standard deviation (±SD) was calculated for every quantitative variable which included the age of the patient, time since the use of metformin, the dosage of metformin use, blood vitamin B12 levels, and glycosylated hemoglobin. The frequency along with percentages was calculated for qualitative variables which included a sexual category and blood vitamin B12 deficiency. A P-value which was below 0.05 was regarded as statistically significant.

## Results

A total of 209 patients was included and enlisted in our study with age group of 45 to 91 years old with a mean age of 66.49±13.35 years, in which 114 (54.5%) were male and 95 (45.5%) were female. Table 1 shows demographic data of the patients.

**Table 1:** Table showing characteristics of patients in our study

Age	66.49±13.35
Gender	
Male	114(54.5%)
Female	95(45.5%)
Duration of diabetes mellitus (Mean, SD)	9.16±5.59
BMI (Mean, SD)	29.43±5.01
HbA1C (Mean, SD)	8.0±2.88
Smoking	80(38.3%)
Hypertension	49(23.4%)
B12 level (Mean, SD)	432±240
Folate level (Mean, SD)	16.9±13.7
Metformin use	122(58.4%)
Metformin dose	
1-1000 mg	12(5.7%)
1001-1999mg	52(24.9%)
>2000mg	58(27.8%)
Acid blocker use	94(45%)
Multivitamin use	83(39.7%)

Serum vitamin B12 level ranged from 122 to 2034 pg/mL. About 53 (25.4%) patients had serum vitamin B12 level less than 150 pg/mL while 63 (30.1%) had an intermediate level between 150 and 350 pg/mL. Nine of these patients with an intermediate B12 level, resulting in a total of 62 (29.66%) of the patients were diagnosed with a metabolic B12 deficiency.

Metformin was used by 122 (58.4%) of patients in our study in which majority 58 (27.8%) were at a dose of > 2000 mg. Only 12 (5.7%) patients were at a dose of 1000 mg or less and the rest 52 (24%) were between 1000 to 2000 mg.

About 94 (45%) of the patients in our study group used acid blockers, mostly in the form of proton pump inhibitor (PPI). Almost 83 (39.7%) of the patients were using multivitamins.

On average, the body mass index (BMI) of the patients with B12 deficiency is almost same as that of the patients without B12 deficiency (p=0.22) 

Same is for HbA1c (p=0.31). The patients with vitamin B12 deficiency suffered from diabetes mellitus significantly for a longer time than patients without vitamin B12 deficiency (p=0.04). The patients on metformin with vitamin B12 deficiency were significantly higher than non-vitamin B12 deficiency (p=0.04). Additionally, in the patients using multivitamins, vitamin B12 deficiency was lower compared to nonusers (p=0.05). In our study, for the patients who smoked, vitamin B12 deficiency was significantly higher than those who did not smoke (p= <0.001). Table 2, showing bivariant association with Vitamin B12 deficiency. 

**Table 2:** Table showing bivariate association with vitamin B12 deficiency

Variable	B12 deficiency		P value
	Yes(n=62)	No (n=147)	
Body Mass Index	29.21±5.01	29.54± 6.5	0.22
HbAIc	8.97±1.65	7.54±2.76	0.31
Length of diabetes mellitus (years)	11.04±6.3	8.54±5.43	0.04
Use of metformin (years)	10.34±5.8	8.39±4.76	0.001
Acid blocker			
Yes	29(46.77%)	65(44.21%)	0.99
No	33(53.22%)	82(55.78%)	
Multivitamin use			
Yes	22(35.48%)	61(41.49%)	0.05
No	40(64.51%)	86(58.50%)	
Smoking			
Yes	39(62.90%)	41(27.89%)	<0.001
No	23(37.09%)	106(72.10%)	

**Figure 1:**
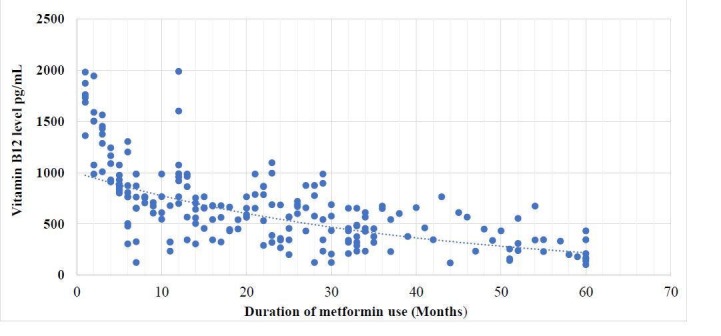
Graph showing the relation of metformin use and vitamin B12 level

We performed a T-test in order to know if any variation was present in the serum vitamin B12 levels of the patients who were using metformin at the time and those who didn’t. Those individuals who were on metformin had comparatively lower levels of serum vitamin B12 and their P value was 0.01. Figure 1 shows the relation of metformin use and vitamin B12 level.

## Discussion

Our cross-sectional study consisted of 29.66% patients with type II diabetes mellitus (T2DM) in the study group who had vitamin B12 deficiency. Matching our acquired prevalence with that of earlier researchers is not straightforward and direct, therefore several factors should be considered. Preceding researchers have concluded comparable results, but on the other hand, the exact mechanisms causing this deficiency have not been well established [10-12].

Inadequate records are available in this regard. Similarly, no study has ever been conducted in Pakistan to evaluate the frequency of vitamin B12 deficiency occurring in diabetic patients prescribed metformin. A previous study conducted by Jager and colleagues [7] which were a randomized controlled trial followed type II diabetes taking metformin 2550 mg/day for 4.3 years concluded that compared to placebo, metformin usage was accompanied by a usual drop in vitamin B12 levels by 19% (95% confidence interval -24% to -14%; P <0.001).

A factor which was considerably linked with vitamin B12 deficiency was determined and included: concurrent intake of metformin with proton pump inhibitors or H2-blockers. The connection of vitamin B12 deficiency and proton pump inhibitors or H2-blockers usage back the concept that decreased gastric acidity plays a vital role as a disposing cause for vitamin B12 malabsorption. Both these medications cause a reduction in acid discharge by the parietal cells and the gastric acid formed by these cells is essential for the breakdown of vitamin B12 from nutritional sources [13-15]. This relationship is very rarely found [16-17]. For example, previously a research conducted by Nervo, et al. [18] found no connection between serum vitamin B12 levels and usage of omeprazole. On the other hand, bearing this in mind the probable collective consequence among metformin and PPI and/or H2-blockers relative to vitamin B12 uptake, caution is advised when these different medications are used in combination.

The patients on long-term metformin treatment are at a greater risk for vitamin B12 deficiency. This study, therefore, proposed that vitamin B12 deficiency must be sought out in Type II diabetes patients on metformin and suggested daily use of multivitamins in order to prevent vitamin B12 deficiency [16]. The use of multivitamins seemed to have a protective role for diabetic patients, preventing vitamin B12 deficiency. Literature documentation suggests the role of multivitamins causing raised levels of serum vitamin B12. Randomized controlled trials conducted on adults who took six to nine micrograms of vitamin B12 on regular basis showed increased levels of vitamin B12 in their blood.

Matching our attained prevalence to studies conducted previously is not upfront and simple with placebo [19]. Also, no studies have been conducted specifically for evaluating whether a multi vitamin on daily basis prevents the occurrence of vitamin B12 deficiency. The findings of our study are notable in the way that typical management of vitamin B12 deficiency is mainly done with greatly increased dosages of supplementations administered orally or parenterally. However, preparations of multi vitamins typically have six to 25 microgram of added vitamin B12 and are sufficient enough for prevention of vitamin B12 deficiency. Additional researches and studies are needed for supporting multi vitamin usage to avoid vitamin B12 deficiency. Apart from this, other possible risk factors known to result in a B12 deficiency, such as progressing age and the use of acid blockers were not considerably found to be associated with vitamin B12 deficiency.

We regard this as a statistically substantial percentage of vitamin B12 deficiency and as a valuable manual for the clinicians to consider it a vital element in the patients with diabetes, predominantly when there has been the use for longer durations and significantly higher dosage of metformin. Though the exact medical importance along with the effect of insufficiency is unidentified, some have proposed that there may very well be an important risk factor for precipitation and/or deterioration of neuropathies along with anemias in a population who are already susceptible to these complications due to the presence of underlying co-morbid diabetes. Prior researchers have revealed neuropathic pains can be avoided with the prescription of vitamin B12 supplements [20-21].

Several limitations have been faced in our study. The first concern was external validity because the research study was performed on a study group in a single-center, the results may be significantly different and considerable variation may exist from the typical diabetic patients present throughout the community. Secondly, this study did not include measurement of methylmalonic acid levels in the blood, which could have resulted in further enhancement of sensitivity by recognizing the vitamin B12 deficiency during its primary non-symptomatic period. Follow-up for determining the outcome of vitamin B12 replacement was also not done. Therefore, a regular follow-up of these patients would have been helpful to evaluate the outcome of the dosage and the time period for which vitamin B12 supplement may be necessary.

## Conclusions

This cross-sectional study confirms that in the patients with type II diabetes mellitus, long-term treatment with metformin and concomitant use of acid blocker are associated with higher chances of developing biochemical vitamin B12 deficiency. Medical doctors, therefore, have to keep this in mind and distinguish it from other factors and therefore need to screen diabetic patients who are on metformin remedy for any secondary vitamin B12 deficiency and primarily patients who come with distressing neurologic symptoms. Moreover, vitamin B12 deficiency can be prevented with a regular intake of added multivitamins or supplements.
